# Current News and Opinion

**Published:** 1886-02

**Authors:** 


### OBITUARY.

### DR. S. C. BARNUM.

Died, December 24th, 1885, at Monticello, Sullivan Co., New York, Sanford C. Barnum, D. D. S., in the 47th year of his age.

In Oakland Valley, a lovely spot, amid forest-clad hills and the grandeur of mountain chains, was born the subject of this sketch. He was a graduate of the New York Dental College, and its honors were never more worthily conferred.

Dr. Barnum for many months was a great sufferer from general debility and nervous prostration. His disease was difficult of diagnosis, and involved in mystery, but the rest that comes to all was his at last, and his strifes are ended

Modest and retiring by nature, he gained the esteem and respect of his profession; skillful and faithful in the field of his labors, the cunning of his hand and the kindness of his heart will long be remembered with admiration and gratitude.

Dr. Barnum entered my office as a student in 1858, and four years later went to practice in the village of Monticello. It was during his stay there that the rubber dam, for drying cavities, was first used by him.

In 1864 he returned to my office as professional assistant, and immediately imparted to me the discovery he had made. At a meeting of the New York Dental Society, in June of that year, he authorized me to present it, a free gift, to the profession. From this resulted a vigorous bound in dental progress, and the name of Barnum became synonymous with bounty and blessing. Dr. Barnum had been the recipient of many substantial and grateful expressions from his contemporaries, when (thirteen years after his beneficent gift) a claim for priority of discovery was set up by another. With health already impaired by a large and exhausting practice, he felt this to be an attack upon his sincerity and well earned fame. It was ill advised and untimely, and sank to the very springs of his life, for its influence he could not control. It is sad and strange that in this benefaction was a peril to him who bestowed it; that through physical weakness and an over sensitive mind, the friend that has left us has won the martyr's crown. J. W. Clowes.

---

### DR. S. G. MARTIN.

Dr. S. G. Martin died at Syracuse, N. Y., Dec. 9, 1885, of Bright's Disease, in the 56th year of his age.

Dr. Martin was born in the town of McDonough, Chenango Co., N. Y., April 11, 1830. He began the practice of dentistry in Meadville, Pa. In 1860, he removed to Syracuse, where he practiced successfully for twenty years, when, on account of ill health, he was obliged to abandon the profession. He was at one time a member of the Fifth District Dental Society, and was the first President of that organization.

C. J. Peters.

DR. ROBERT L. ROBBINS.

While on his way to the Cambridge station on the Fitchburg railroad, on Dec. 29th, Dr. R. L. Robbins, of Boston, dropped upon the sidewalk, dying before assistance could be rendered him. His death was attributed to heart disease. He was about 65 years of age, and had lived in Boston nearly a half a century, having moved there from East Cambridge when a small boy. Dr. Robbins was widely known in the dental profession, and had been the treasurer of the Boston Dental College from its organization. He was much respected by a large circle of patients, friends and neighbors. A. M. D.

UNIVERSITY OF CALIFORNIA.

The fourth annual commencement exercises of the College of Dentistry of the University of California were held in connection with those of the Medical Department, at the Grand Opera House, San Francisco, Tuesday, November 10th, 1885, at 2 o'clock P. M.

The number of matriculants for the session was thirty-seven.

The degree of Doctor of Dental Surgery was conferred upon the following graduates:

Harry Sylvester Bettis,

George Botsford,

Daniel Barratt Cate,

Nathaniel Thomas Coulson,

George Ihnier Drucker,

William Ellis Fitzpatrick,

Walter Robert Henderson,

John Adams Douglass Hutton.

Franklin Pancoast,

Charles Theodore Rodolph,

Frederick Judson Saxe, A. M.

Joseph Schneider,

Henry Sylvester, Jr.

ST. LOUIS DENTAL SOCIETY.

At the annual meeting of the St. Louis Dental Society, held Jan. 5th, 1886,
Dr. Wm. N. Conrad, was elected, *President.*
Dr. Geo. A. Bowman, *Vice-President.*
Dr. A. H. Fuller, *Corresponding Secretary.*
Dr. J. L. Foster, *Recording Secretary.*
Dr. H. H. Keith, *Treasurer.*

A. H. FULLER, *Recording Secretary.*

HONORS FOR HERBST.

At the meeting of the Central Verein Deutscher Zahnarzte, held at the city of Nurnberg, August the 3d, 4th and 5th, 1885, Dr. Wm. Herbst, of Bremen, in recognition of the services rendered to the dental profession, was presented with a gold medal.

At the meeting of the Association generale des dentistes de France, and the Societe civile de l'Ecole et de l', hopital dentaires de Paris, held October 20th, 1885, Dr. Wm. Herbst was unanimously elected to honorary membership.

At the meeting of the Societe d' Odontologie de Paris, held in October, 1885, Dr. Wm. Herbst was elected a corresponding member.

### MASSACHUSETTS DENTAL SOCIETY.

The twenty-first annual meeting of the Massachusetts Dental Society was held at the hall of the S. S. White Dental Manufacturing Co., No. 160 Tremont St., Boston, on Thursday and Friday, Dec. 10 and 11, the President, Dr. J. F. Adams, of Worcester, in the chair. The annual address was delivered by Dr. D. B. Ingalls, of Clinton, Mass. Dr. W. H. Atkinson, of N. Y., read a paper on the "Relation of Special to General Practice." Dr. David Hunt, of Boston, the eminent oculist, read a paper entitled "An Influence Affecting the Development of the Lower Jaw." Dr. H. C. Merriam, of Salem, Mass., spoke upon "The Preparation of Roots for Filling." Dr. J. N. Farrar, of N. Y., read a paper entitled "Blackboard Illustrations and Chalk Talks."

All of these subjects were discussed by different members of the profession. We expect to publish several of these papers in future issues of the INDEPENDENT PRACTITIONER.

The following were elected officers for the ensuing year:

*President.*—Dr. S. G. Stevens, Lynn.

*Vice-Presidents*— { Dr. E. B. Hitchcock, Boston.<br>Dr. H. C. Merriam, Salem.

*Secretary*—Dr. W. E. Page, Boston.

*Treasurer*—Dr. E. Page, Charlestown.

*Librarian and Microscopist*—Dr. R. R. Andrews, Cambridge.

### EXECUTIVE COMMITTEE.

Dr. G. F. Eames, Boston.
Dr. J. G. W. Werner, Boston.
Dr. J. K. Knight, Hyde Park.
Dr. E. C. Leach, Boston.
Dr. F. A. Cooke, Boston.
Dr. R. R. Andrews, Cambridge.

### ILLINOIS STATE DENTAL SOCIETY.

The Executive Committee of The Illinois State Dental Society announces the following list of reports and essays for the next annual meeting, which will be held at Rock Island, the second Tuesday in May.

1.—Report of the Committee on Dental Science and Literature,—
Dr. HOMER JUDD, of Alton, (Chairman).

2.—Report of the Committee on Dental Art and Mechanism,—
Dr. J. FRANK MARRINER, of Ottawa, (Chairman).

3.—Essay, Dr. JOHN S. MARSHALL, of Chicago,—"Oral Surgery."

4.—Essay, Dr. C. R. TAYLOR, of Streator,—
"Preparation of Pulp Canals and Cavities for Filling."

5.—Essay, DR. HOMER JUDD, of Alton,—
"The Retention of Pulpless Teeth in the Jaws."

6.—Essay, Dr. J. D. MOODY, of Mendota,—"Post Graduate Study."

7.—Essay, Dr. J. G. REID, of Chicago,—"Oral Chemistry."

8.—Essay, Dr. E. S. TALBOT, of Chicago,—

"Separating Teeth Preparatory to Filling Them."

It is probable that Dr. G. V. Black, of Jacksonville, may have an essay, the subject of which he is not yet ready to announce.

Edmond Noyes,
E. J. Green,
W. H. Taggart,
Executive Committee.

MARRIED.

In New York, January 21st, 1886, at the residence of the bride's father, Meyer L. Rhein, M. D., D. D. S., to Lizzie Esse, daughter of Dr. F. M. Odell. The many friends of the contracting parties wish them all happiness, and that their married life may be long and prosperous.

The Louisville Medical News and *The American Practitioner* are to be consolidated under the name of *The American Practitioner and News.* The new journal will be published bi-weekly, and it will be edited by D. W. Yandell, M. D., of *The Practitioner*, and H. A. Cottell, M. D., of *The Medical News.*

If we are to have all the virtues of both these excellent journals condensed into one, it will make a model indeed. It looks like it, when Drs. Yandell and Cottell become the editors, for both are men of undoubted literary ability, and both are possessed of wide experience and thorough training. The new journal will be published at $3.00 per year. Address John P. Morton & Co., Louisville, Ky.

The Latest Device in office thieving is founded upon the habit which ladies have of carrying their purse in a cloak pocket or bag. A well dressed rascal waits until he sees such an one enter an office when he is certain that no other patients are present. He then enters and requests that some trifling operation be performed. But he is in no hurry; he will wait till the dentist is at liberty. In the meantime he watches for his opportunity, ransacks the cloak pockets or reticule of the patient in the chair, and slips out, either silently or under some pretense. Look out for him. The offices of a number of dentists in Buffalo have been thus robbed.

Dr. R. I. Pearson, of Kansas City, has purchased the Kansas City Dental Depot, and will henceforth be the head of the house of R. I. Pearson & Co. Dr. Pearson is very widely and as favorably known to the dentists of the south-west, and they now have the opportunity to manifest their high regard for him in a substantial manner. His interest in dentistry has always been exceedingly active, and he has been the earnest supporter of dental societies and meetings. He was formerly the very efficient editor-in-chief of *The Missouri Dental Journal.* His eastern friends wish him the most abundant success.

The retirement of Oakley Coles from dental practice is to be signalized by the presentation of a testimonial. A committee to raise the necessary funds and to arrange the affair has been appointed, of which Sir Edwin Saunders is chairman, and Mr. Charles Varey is treasurer.

Dr. E. M. Nelson, of Lowell, Mass., has been arrested for complicity in the robbing of the Lancaster National Bank, of Mass., by its president, Mr. Wm. H. McNeil. Dr. Nelson formerly enjoyed a large practice, but becoming interested in various speculations he had neglected his business, and finally retired from practice altogether. He was interested in the West Rutland Marble Company, whose doings lately absorbed so much of public attention, and whose losses, it is charged, were made good by peculations from the Lancaster bank.

---

Dr. C. Edmund Kells, Jr., advises that when an artificial nose is fashioned of hard rubber, the edges which come in contact with the tissue of the face should be made of soft velum rubber. If the hard rubber be in contact during the muscular movements of talking or smiling, a clear line of demarcation between the movable tissue and the immovable nose will be seen. But when the velum rubber is used at the edge, the motions are lost in it and the line is not observable.

---

An appreciative friend, who is imbued with the proper spirit, writes as follows: "I have an article for the Independent Practitioner which I will forward at an early date, with the complete understanding that you shall change, criticise, transpose, cut down or reject altogether, as in your judgment may seem best; in other words, you shall carry out Dr. Cushings' idea of the editorial privilege and duty to its fullest extent, for I am satisfied that he is correct."

---

Pasteur is now constantly receiving hydrophobic patients from all over the world. A number have gone to him from the United States. The process consists essentially in inoculation with the attenuated virus of rabies, which has been reduced in potency by being passed through a number of organisms. If the period of incubation of the disease has not passed, this is supposed to be as perfect protection as is vaccination for small-pox,

---

Conditions which are in nowise connected with the reception and absorption of lime salts, such as bad hygiene of dwellings, syphilis, etc., favor the development of rachitis in a most remarkable degree. The deficiency of lime in rachitic bones is called forth, singly and alone, by local inflammatory process. But the local process in the bones in turn has its origin in some preceding anomalous condition of the entire organism.—*Phil. Med. Times.*

---

The Polyclinic gives a test for determining the character of the discharge from a suspected salivary fistula. It consists in bringing a drop of the fluid into contact with a drop of the tincture of chloride of iron on a white surface, like a sheet of paper, when if the discharge contains saliva it will give a pink color, thus indicating the presence of the sulpho-cyanide of potassium, an ingredient of normal saliva.

---

The Medical Record gives an acconut of a case in which the two lower central incisors were found perfectly erupted at birth. Since that time the other incisors have made their appearance, but are smaller than the first ones.

A SINGULAR ACCIDENT occurred in the practice of J. J. H. Sanders, of Barnstaple, England. In attempting to remove a superior bicuspid tooth, the palatine blade of the forceps broke and went down the trachea. Suffocation at the time was avoided by inversion, but secondary trouble manifested itself some time after, and the foreign body was finally removed by tracheotomy.

---

THE FRENCH have a saying that is not very complimentary to our profession, but which doubtless had its origin in the time of the notorious traveling charlatans who made such extravagant pretentions—*Il est menteur comme un arracheur de dents.* He is as great a liar as a dentist; he has infinite conceit in his own professional skill.

---

ANÆSTHETICS caused the death of eighteen persons in England and Scotland last year. Nine were from the use of chloroform, six from ether, and three from a mixture of ether and chloroform. In every fatal case the patient had been comparatively healthy, and the operation was of a slight character.—*Caulk's Dental Annual.*

---

THE QUARTERLY COMPENDIUM says that while fossil human teeth contain large quantities of fluorine, the teeth of to-day show but very little in their composition.——Is this assertion founded upon any reliable analysis, or is it merely conjectural? It should not be received until positively proved. Who knows anything about it?

---

THE COURSE OF STUDY for a graduate in medicine in Belgium extends through seven years; in Russia, five years; in Denmark, seven years; in France, four years; in Italy, six years; in Germany and Austria, about four years, but the graduate must have a certificate of graduation from a public gymnasium or high school.

---

DR. A. W. HARLAN, of Chicago, sailed for Liverpool on the 7th ult., for a brief visit to London, Paris, and Berlin. Insomnia had claimed him for a victim, and an ocean voyage was recommended. His many friends will earnestly hope that the trip may be beneficial. He will return the last of the present month.

---

WE WONDER if many of our readers have tried the small brushes that are adapted to the dental engine? They render good service in polishing stains from the teeth, and cost so little that there can be no excuse for using them a second time. They should be charged with powdered Arkansas stone moistened with glycerine. C. E. F.

---

MURIATE OF COCAINE, if we can believe the assertion of observers, is the most seductive and dangerous drug which has ever been abused by man. The Cocaine habit, when once established, is far more debasing and destructive than is that of Opium or Alcohol.

---

COCAINE is a Methylbenzomethoxyethyltetrahydropyridinecarboxylate. Why is it not called by its proper name?

THE MEDICAL ANALECTIC, that excellent journal published by G. P. Putnam's Sons, will in future be under the editorial managment of Dr. B.W. Amidon. New and more complete arrangements have been made for the reception of foreign journals, from which abstracts and translations will be made.

---

THE BUFFALO CREMATORY has been completed and the first body incinerated. The test was very successful indeed, the whole process occupying only about an hour, and the results even better than could have been anticipated. The building is a beautiful one, and all the accessories are in keeping.

---

A VERY FEW of our exchanges come to us rolled up so closely that it is very difficult to get them open without tearing. A publisher who has no more respect for his journal than to roll it up like a bandage, does not deserve to have it read or respected by any one else.

---

M. DUCHESNE, a French advertising dentist, has lately been condemned by the civil courts to pay a fine of 600 francs, and 3000 francs damages to the family of a patient who died under the administration of nitrous oxide given at his hands.

---

ERRORS WILL appear in the most carefully prepared reports. The remarks ascribed to Dr. Harlan, on page 28 of the January number, were really made by Dr. Marshall. "Dr. Davidson," on the same page, should read, Dr. Davis.

---

JOHN C. DRAPER, M. D., L. L. D., Professor of Chemistry in the Medical Department of the University of the City of New York, died in that city after a brief illness, on the 20th of December last.

---

DR. E. PARMLY BROWN is always presenting something new. His latest is a polishing point for the dental engine, made of Moose hide, and intended to carry polishing powder. It is very effectual.

---

PROF. VIRCHOW says that "no specialty can really flourish which cuts itself entirely off from the great body of the science." How is it when the science cuts itself off from the specialty?

---

THE MEDICAL SOCIETY OF THE STATE OF NEW YORK will hold its eightieth annual meeting in the Common Council Chamber at Albany, Feb. 2, 3, and 4, 1886.

---

THE DETROIT LANCET will henceforth be known as *The American Lancet.* It will be quite as readable and instructive under the new name, and that is saying a great deal.

---

PROF. R. OGDEN DOREMUS reports to the New York Medico-Legal Society a case of death from the application of Cocaine to relieve the pain from a decayed tooth.

